# Falling Into a Trap

**DOI:** 10.1007/s40670-020-00968-3

**Published:** 2020-04-28

**Authors:** David Walsh

**Affiliations:** Medical College of Georgia at Augusta University, Augusta, GA USA

While rounding with our team, I brought up a case I had come across recently. I described the complex case to the team, ultimately leading to a bone marrow biopsy that was TRAP stain positive. Admittedly, I had missed the diagnosis on the first pass. Inquiring into our team’s knowledge of the use of a TRAP stain, the resident was stumped. Additionally, he appeared slightly annoyed that I was asking such an esoteric, “zebra-esque” question. But the enthusiastic intern’s mind was working. She was processing the information and digging deep. She first said, “A TRAP stain? That is for cancer or that is for leukemia. Cutaneous T-Cell lymphoma, no that’s not it. Oh! I remember!” she exclaimed. “That’s for Hairy Cell Leukemia.” I congratulated her on her medical knowledge.

Later in the day, we met a new patient. He had presented to the hospital last night for abdominal pain and early satiety. He had been to multiple ERs over the course of the past few weeks for similar complaints. He had seen multiple outpatient providers prior to this admission. The patient was upset related to his symptom management. The type of upset resulting in the admitting night team being upset, the nurses were upset, and the charge nurse was upset. Our collaborative rounds with nursing, pharmacy, and case management usually occur at the bedside. I had been “warned” that it may be better to discuss this patient outside due to his known ongoing substance abuse. The team proceeded to tell me about the difficult interaction with the patient. The same intern that answered the question earlier recalled about how she spent 30 min just pre-rounding on him. She described behavior consistent with noncompliance and one pursuing secondary gains. After collaborative rounds, the resident, intern, and I went to evaluate the patient.

Our interaction started as one might expect. Our patient showed behavior consistent with his known substance abuse problem and became increasingly agitated as the conversation progressed. Stepping back—while observing my team—the first thing that I noticed was that both intern and resident stood in a manner that showed respect for the patient. Their body language provided affirming nonverbal cues. Their dress was professional. They were actively listening to the patient. They provided the patient time to express his frustrations. They made eye contact with the patient. They remained calm, despite escalation on the part of the patient. Then, the patient paused and fell quiet.

After a purposeful period of silence, the intern succinctly explained the plan of care for the day. She showed empathy for the patient’s pain without compromising her plan on an appropriate non-narcotic pain control regimen. The resident thanked the patient for sharing his frustration. He proceeded to describe to the patient the complexity of the situation and acknowledged the frustrations that are often associated with cases similar to his. He did not use inflammatory or dismissive phrases such as, “we don’t think there is anything wrong with you.” But instead, he described the multiple possible diagnosis that could be the etiology of his symptoms. He concisely described what the next steps were. He was able to re-direct when the discussion went off track.

In retrospect, the two physicians in training provided a therapeutic encounter without prescribing any medications or definitive diagnosis. I did not need to say a word and only thanked the patient for letting us care for him as I was leaving the room.

When we left, I stopped the team and wanted to make it a point to give positive feedback. We talked about how they just demonstrated excellence in patient care. Specifically, through their use of communication skills and professionalism, they were able to develop patient-physician trust and show empathy. They sustained that trust when disagreement about the plan of care arose between the patient and care team. No diagnosis was given, but the foundation for ongoing evaluation was provided. In this educator’s opinion, they were aspirational in these domains. They both thanked me. The resident, whom is a trained educator as well, noted that they rarely get feedback on these matters. I pried a bit deeper.

“Did you learn to do this well as part of your training during your Master’s in Education?”

“No.” He replied

“Was it read or taught in a memorable way.”

“Not that I can recall.”

“Then how?”

“I guess by watching physicians I respect and just thinking about how people should be treated.”

I am aware and proud of the strength of our residency program and the emphasis on the striving to teach the ACGME core competencies. I am continually impressed with the way in which our residents live out these competencies and become smart, competent chief residents, fellows, and practicing physicians. But I cannot help to think that we as educators still have work to do, both locally and nationally. We are usually very strong at evaluating medical knowledge and do so freely [6]. I wonder if we as an educational community still shy away from the other competencies. This, despite the public’s expectation of the development of these skills [9]. Data would suggest this to be the case [1–5]. Further, evidence is emerging that educators continue to use an overall “gut feeling” about residents despite efforts by ACGME and others to further delineate performance metrics [10]. Systems hold responsibility as well. Despite the ACMGE providing more comprehensive tools to define deficiencies, 22% of accredited programs were recently found to not have an adequate method in place to capture those deficiencies [7]. This begs the question is it enough to rely on emulation and “gut feelings”? But, by an overwhelming majority, residents appear to learn an acceptable requisite of these skills as evident by residency graduation rates. This author’s hypothesis is that it is self-taught, maybe with the assistance of learning through passive observation of behavior modeled by their peers, fellows, and attending physicians. While efforts are ongoing, research is still needed in this area to provide granular, usable, and actionable tools for educators [4, 8]. All said, a fundamental question remains. If we have limited opportunities on a given day to give feedback on the core competencies, are we still spending the majority of those fleeting opportunities on medical knowledge assessment?

After rounds, I light-heartedly asked, “So are you happier that I gave you feedback on your patient encounter or that you knew the diagnostic value of a positive TRAP stain?” The intern smiled and said, “Oh the TRAP stain for sure.”
